# Clozapine-induced acute gastrointestinal necrosis: a case report

**DOI:** 10.1186/s13256-017-1447-4

**Published:** 2017-09-23

**Authors:** Mark T. Osterman, Caitlin Foley, Isaac Matthias

**Affiliations:** 1 Division of Gastroenterology, Perelman School of Medicine, University of Pennsylvania, Philadelphia, Pennsylvania USA; 2 Section of Hospital Medicine, Perelman School of Medicine, University of Pennsylvania, Philadelphia, Pennsylvania USA

**Keywords:** Clozapine, Quetiapine, Antipsychotic, Acute gastrointestinal necrosis, Acute bowel necrosis, Acute esophageal necrosis

## Abstract

**Background:**

Clozapine is known to cause fecal impaction and ileus with resultant colonic necrosis due to compression of colonic mucosa. There are rare reports of clozapine causing necrosis of other portions of the gastrointestinal tract unrelated to constipation. We describe a case of acute necrosis of the upper gastrointestinal tract and small bowel to due to clozapine and quetiapine.

**Case presentation:**

A 66-year-old white man with a past medical history of schizophrenia, maintained on clozapine and quetiapine, presented with hypoxic respiratory failure caused by aspiration of feculent emesis due to impacted stool throughout his colon. His constipation resolved with discontinuation of clozapine and quetiapine, and his clinical condition improved. These medicines were restarted after 2 weeks, resulting in acute gastrointestinal necrosis from the mid esophagus through his entire small bowel. He died due to septic shock with Gram-negative rod bacteremia.

**Conclusions:**

Clozapine may cause acute gastrointestinal necrosis.

## Background

Constipation is a common side effect of antipsychotic medicines, and is attributed to their anticholinergic properties [1]. Constipation due to antipsychotics can progress to severe pathology such as ileus, fecal impaction, aspiration of feculent emesis, and colonic ischemia due to compression of colonic mucosa [2]. Life-threatening complications of constipation due to antipsychotic medication occur most frequently with clozapine, and are estimated to occur in 0.3% of patients who receive this medicine [3, 4]. It is not certain whether clozapine can cause ischemia in portions of the gastrointestinal tract other than the colon, unrelated to constipation. We present a case of a patient who developed acute necrosis of the esophagus, stomach, and small bowel shortly after clozapine re-initiation.

## Case presentation

**Fig. 1:**
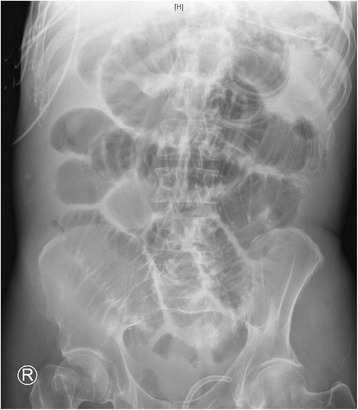
Abdominal X-ray showing diffuse small bowel dilation

**Fig. 2:**
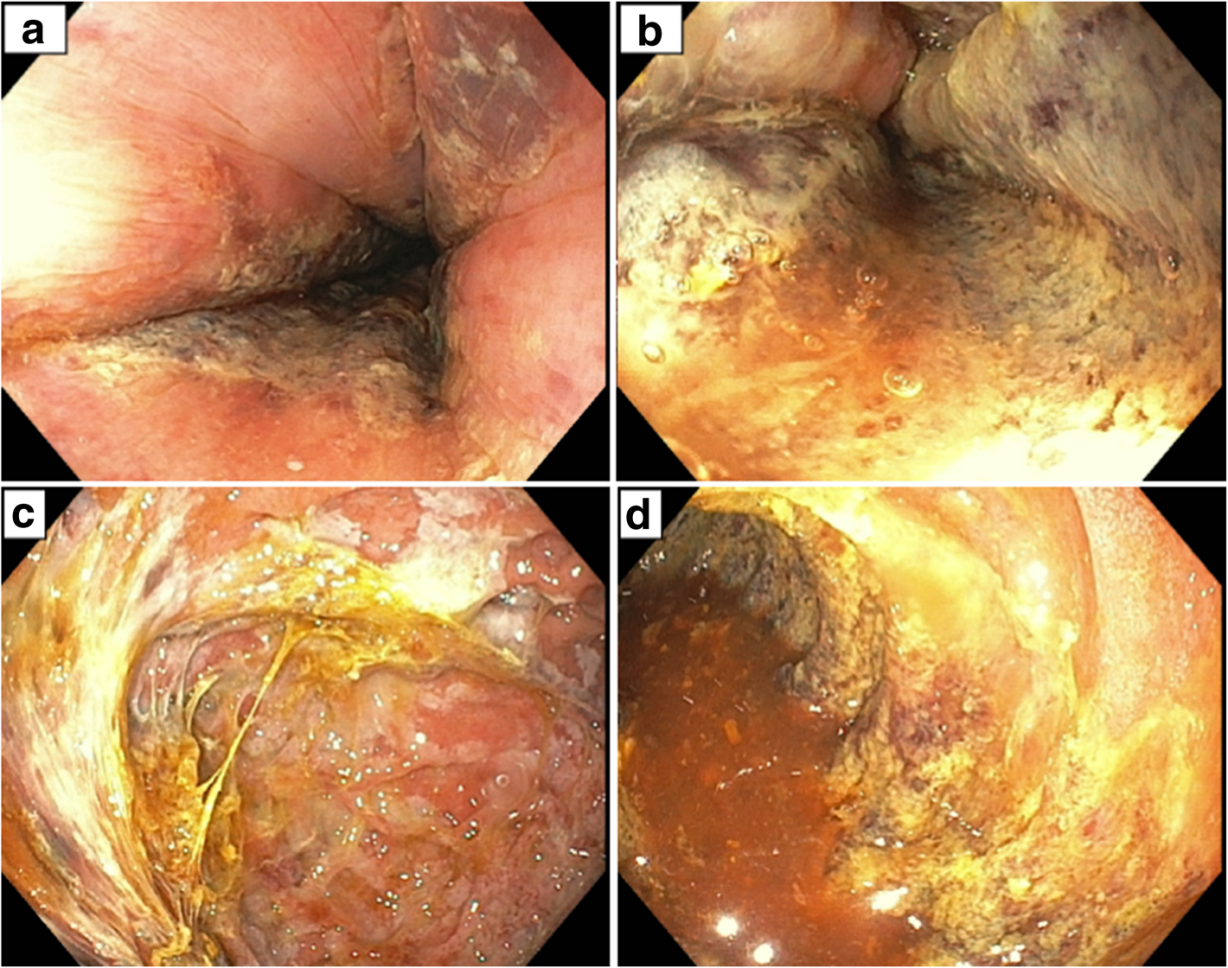
Esophagogastroduodenoscopy images showing severe diffuse ulceration of the upper gastrointestinal tract. **a** Transition from normal to ulcerated mucosa in the mid esophagus, **b** distal esophagus, **c** stomach, and **d** duodenum

A 66-year-old white man with a history of schizophrenia presented to our emergency department with acute altered mental status and feculent emesis. His abdomen was distended but not tender. He was found to be in severe respiratory distress requiring endotracheal intubation, and shock requiring vasopressor infusion. Computed tomography (CT) imaging revealed acute respiratory distress syndrome (ARDS) due to aspiration as well as impacted stool throughout his colon. Home antipsychotic medicines, clozapine and quetiapine, were held, and after manual disimpaction and enemas he had copious bowel movements and resolution of abdominal distension. His shock and ARDS gradually improved. Vasopressors were discontinued on day 3, and he remained normotensive. In-patient medicines were carbamazepine, lansoprazole, phenytoin, polyethylene glycol, senna, and subcutaneous heparin prophylaxis. Clozapine 100 mg every 12 hours was restarted on day 11, and a single dose of quetiapine 400 mg was given on day 16, both at home doses. Six hours after quetiapine was restarted, he was noted to have recurrence of abdominal distension and new onset of copious foul-smelling hematemesis. An abdominal X-ray showed diffuse small bowel dilation (Fig. 1). An urgent esophagogastroduodenoscopy (EGD) was performed, showing transition from normal mucosa in the proximal esophagus to severe diffuse ulceration beginning in the mid esophagus (Fig. 2a), with continued severe ulceration throughout the distal esophagus (Fig. 2b), stomach (Fig. 2c), and duodenum (Fig. 2d). He developed progressive septic shock, and blood cultures ultimately grew *Escherichia coli* and *Klebsiella oxytoca*. Surgical consultation was obtained, but no surgical intervention was possible given the widespread gastrointestinal involvement and his moribund state. He died within 24 hours of developing symptoms. An autopsy was declined.

## Discussion

Our patient’s initial presentation with aspiration of feculent emesis due to impacted stool throughout the colon is within the known spectrum of life-threatening clozapine-induced colonic hypomotility. Fecal impaction resolved with discontinuation of clozapine and quetiapine.

Acute gastrointestinal necrosis developed 5 days after restarting clozapine and 6 hours after restarting quetiapine. EGD and abdominal X-ray were consistent with necrosis from the mid esophagus through the entire small bowel. His new onset foul smelling hematemesis, abdominal distension, and polymicrobial Gram-negative rod bacteremia clinically matched imaging findings, and he died within 24 hours of developing symptoms. Given the temporal association with his clinical deterioration, we believe his acute gastrointestinal necrosis was caused by re-initiating clozapine and quetiapine.

Other causes of gastrointestinal necrosis were considered. It is unlikely that proximal splanchnic arterial occlusion from embolic or other source occurred, as necrosis was present in multiple vascular territories. Our patient had remained off vasopressors with normal blood pressure for 13 days, making a shock state an unlikely cause. His other medications did not include vasoactive drugs known to cause mesenteric ischemia such as alpha agonists, digoxin, or ergotamines [5].

There are few reports of clozapine causing acute necrosis of portions of the gastrointestinal tract other than the colon. Yu *et al*. in 2013 reported a case of a 47-year-old man who developed acute necrosis of small bowel and colon 6 days after initiating clozapine [6]. Pautola and Hakala in 2016 reported a case of a 65-year-old man who developed acute esophageal necrosis 6 hours after receiving a single dose of clozapine and olanzapine due to medication error [7]. As in our patient, these cases occurred shortly after initiating clozapine, involved portions of the gastrointestinal tract outside the colon, and were unrelated to constipation.

A possible mechanism of clozapine-induced acute gastrointestinal necrosis is antiserotonergic effect. Clozapine is the only antipsychotic medicine known to be a competitive inhibitor of the 5-hydroxytryptamine_3_ (5-HT_3_) serotonin receptor [8]. Alosetron, a potent 5-HT_3_ inhibiting drug used to treat irritable bowel syndrome with diarrhea, was withdrawn from the market due to causing ischemic colitis unrelated to constipation, and was subsequently reintroduced with a black box warning [9, 10].

## Conclusions

Clozapine is known to cause fecal impaction and ileus, compressing the colonic mucosa and leading to colonic necrosis. Two recent cases have described clozapine-induced acute necrosis of the small bowel and upper gastrointestinal tract, without constipation. We present a third such case. Further monitoring is needed to confirm this suspected life-threatening side effect of clozapine.

## Abbreviations

5-HT3: 5-Hydroxytryptamine3
ARDS: Acute respiratory distress syndrome
CT: Computed tomography
EGD: Esophagogastroduodenoscopy
